# Bacterascites caused by *Salmonella* spp. following a cesarean section: A case report

**DOI:** 10.1097/MD.0000000000039017

**Published:** 2024-07-19

**Authors:** Ya-Chu Wu, Wang-Chun Ho, Sheng-Po Kao, Dah-Ching Ding

**Affiliations:** a Department of Obstetrics and Gynecology, Hualien Tzu Chi Hospital, Buddhist Tzu Chi Medical Foundation, Tzu Chi University, Hualien, Taiwan; b Institute of Medical Sciences, Tzu Chi University, Hualien, Taiwan.

**Keywords:** bacterascites, case report, cesarean delivery, preeclampsia, prolonged labor, *Salmonella*

## Abstract

**Rationale:**

Bacterascites are a rare complication of cesarean sections (C/S). Here, we report the case of a patient with bacterascites after an emergent C/S.

**Patient concern:**

A 41-year-old female reported diffuse abdominal tightness and pain for a week after C/S, who received C/S at 38 4/7 weeks due to superimposed preeclampsia and prolonged labor.

**Diagnoses:**

Bacterascites caused by *Salmonella* species after C/S was diagnosed.

**Interventions:**

Initial treatment included cefmetazole and metronidazole. On day 2, paracentesis was performed, followed by albumin and hydroxyethyl starch administration. By day 3, the patient developed pulmonary edema, necessitating Lasix administration. On day 6, ascites culture revealed Salmonella species resistant to third-generation cephalosporins, leading to meropenem therapy adjustment. This resulted in improved symptoms. Meropenem was continued for 14 days to complete the treatment regimen.

**Outcomes:**

Follow-up ultrasonography revealed a decrease in ascites. As the patient clinical condition improved, she was discharged on day 20 and scheduled for outpatient department follow-up. No recurrence of ascites was observed during the subsequent follow-up period of 3 months. No ascites were noted 8 days after discharge.

**Lessons:**

Postoperative bacterascites with *Salmonella* were diagnosed. Antibiotic treatment and therapeutic paracentesis were effective for this condition.

## 1. Introduction

Bacterascites, also known as spontaneous bacterial peritonitis (SBP), is a serious and potentially life-threatening condition characterized by ascitic fluid infection within the peritoneal cavity without an evident intra-abdominal source of infection.^[1]^ The peritoneal cavity is a space in the abdomen that contains organs such as the stomach, liver, and intestines, and normally, it has a small amount of fluid to lubricate the organs and facilitate movement.

SBP typically occurs in individuals with underlying liver cirrhosis, advanced liver disease, or other conditions that lead to the accumulation of ascitic fluid, such as heart failure or certain cancers.^[2]^ In these individuals, ascites weaken the body defense mechanisms within the peritoneal cavity, making it more susceptible to bacterial invasion and infection.^[3]^

The bacteria responsible for SBP are usually of intestinal origin, commonly Escherichia coli, Klebsiella pneumoniae, or Streptococcus species.^[4]^ These bacteria gain access to the ascitic fluid through translocation from the gut lumen, where they usually reside, or through direct hematogenous spread from other infected sites in the body.^[5]^

The clinical presentation of SBP can vary widely, ranging from subtle symptoms such as abdominal discomfort, altered mental status, and low-grade fever to more severe manifestations including abdominal pain, fever, chills, and signs of systemic infection such as hypotension and altered consciousness.^[6]^

Prompt diagnosis and treatment of SBP are crucial to prevent complications such as septic shock, renal failure, and hepatic encephalopathy, which can significantly increase morbidity and mortality.^[6,7]^ Diagnosis typically involves a combination of clinical suspicion, analysis of ascitic fluid obtained through paracentesis, and laboratory tests such as cell count, differential, and culture.

Treatment of SBP typically involves the administration of broad-spectrum antibiotics targeting the most common causative organisms, along with supportive measures to stabilize the patient and address any underlying conditions contributing to ascites formation.^[8]^ In some cases, repeated paracentesis or placement of a peritoneovenous shunt may be necessary to manage refractory ascites.^[9]^

Overall, bacterascites or SBP represents a serious complication of predisposing conditions (e.g., cirrhosis, heart failure, cancer, nephrotic syndrome, and systemic lupus erythematosus), requiring prompt recognition and appropriate management to improve outcomes and reduce the risk of complications.^[1]^ Regular monitoring and prophylactic measures may also be necessary to prevent recurrence in susceptible individuals.

Bacterascites are a rare complication of cesarean sections (C/S). Here, we report the case of a patient with bacterascites caused by *Salmonella* after C/S that was necessary after prolonged labor and superimposed preeclampsia. Written informed consent was obtained from the patient to publish this paper.

## 2. Case report

A 41-year-old female, gravida 1, para 1, with a history of chronic hypertension, underwent C/S due to complications of superimposed preeclampsia and prolonged labor at a gestational age of 38 4/7 weeks. Discharged 3 days post-C/S, she subsequently experienced diffuse abdominal discomfort, intermittent rib area pain, and bilateral lower leg edema.

Upon presentation to the emergency department, a physical examination revealed diffuse abdominal dullness and rebounding pain. Transabdominal ultrasound confirmed ascitic fluid accumulation, prompting admission for further evaluation and management of suspected complicated ascites. The patient had no reported drug or food allergies. Her family history was unremarkable except for her father diabetes mellitus.

Vital signs upon admission included a body temperature of 36.0°C, blood pressure of 146/81 mm Hg, pulse rate of 89/min, and respiratory rate of 19/min. Clinical examination indicated a clean C/S wound without ecchymosis and normal active bowel sounds, alongside rebound pain and dull abdominal percussion. Laboratory findings revealed low total phosphate (5.9 g/dL), low albumin (2.8 g/dL), elevated C-reactive protein (31.59 mg/dL), leukocytosis (16,320/µL), and bacteriuria (WBC, 75–100/high power field), which means infectious status. Paracentesis was performed on day 2, and ascitic fluid culture was positive for Salmonella species. Microscopic examination of ascites showed WBC was 5698/µL and 72% neutrophils. Biochemistry of ascites showed total protein 3.6 g/dL (normal 1.5–3), Albumin 2.0 g/dL (normal > 1.1), and globulin 1.6 g/dL (normal < 0.6), which means inflammatory or infectious conditions. To rule out urine leakage after surgery, the kidney profile of ascites was checked (BUN 12 mg/dL and Creatinine 0.64 mg/dL), which showed no urine leakage.

**Figure 1.**
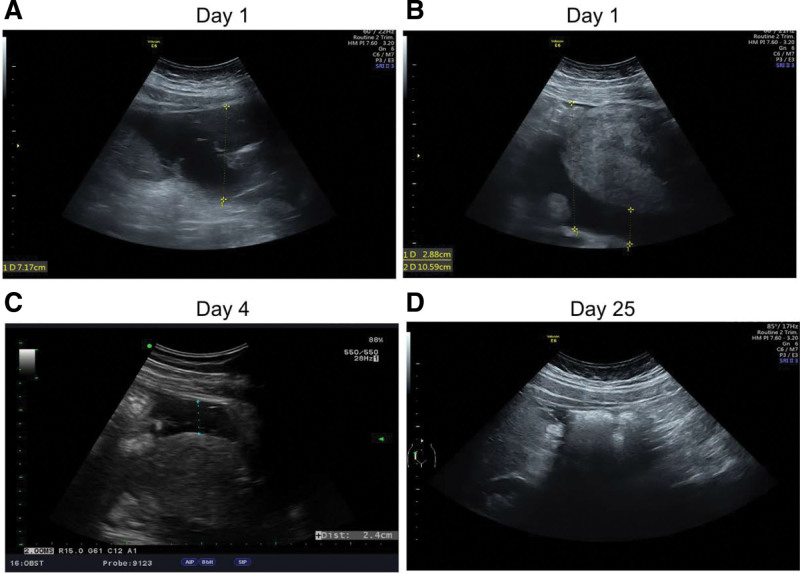
Ultrasound examination of bacterascites. (A) Ascites at the right upper quadrant of the abdomen on d 1. The largest pocket was 7.1 cm. (B) Ascites at the cul-de-sac on day 1. (C) Post-admission d 4. Ascites decreased a lot. (D) Post-admission d 25 (8 d after discharge). No ascites were noted.

Abdominal ultrasonography demonstrated ascites along the gutter and cul-de-sac regions (Fig. 1A and B). Subsequent ultrasonography indicated decreased ascitic fluid volume over time (Fig. 1C and D). Empirical antibiotic therapy with cefmetazole and metronidazole was initiated, with subsequent adjustments (meropenem) due to bacterial resistance after the culture results came out on day 6 and continued to be used for 14 days (suggested by the doctor of the Infection Department). Management also included albumin and hydroxyethyl starch administration for hypoalbuminemia and fluid resuscitation (since day 2), respectively. Anti-hypertensive medications were adjusted to manage persistent high blood pressure.

Complications such as acute pulmonary edema with bilateral pleural effusion necessitated additional interventions on day 3, including diuretic therapy (Lasix 20 mg intravenous injection twice a day) and antibiotic adjustments. The patient responded well to treatment, with gradual improvement in clinical symptoms and resolution of ascites. She was discharged on day 20 with outpatient department follow-up.

During subsequent 3-month follow-ups, no ascites recurrence was noted, indicating successful management of the bacterascites and associated complications.

## 3. Discussion

We report a 41-year-old woman with chronic hypertension underwent C/S due to superimposed preeclampsia. Post-discharge, she developed diffuse abdominal discomfort. Admitted for suspected complicated ascites, tests confirmed Salmonella bacterascites. Treatment included antibiotics, fluid resuscitation, and diuretics, resulting in the resolution of symptoms. Follow-up showed no recurrence, indicating successful management.

This case reports thinking process included recognizing the patient details, including age, gender, obstetric history, underlying medical conditions (chronic hypertension), identification of key symptoms and signs, diagnostic evaluation and interpretation, treatment planning, monitoring, and follow-up.

Empirical antibiotic therapy for bacterascites, also known as SBP, should be chosen based on the likely causative organisms and local antibiotic resistance patterns.^[10]^ The most common pathogens associated with SBP are gram-negative bacteria, particularly Escherichia coli and Klebsiella pneumonia, as well as Streptococcus species.^[1]^ Our case was Salmonella infection, a gram-negative bacilli.

Initial empirical antibiotic choices for SBP often include broad-spectrum antibiotics that provide coverage against these common pathogens. A commonly used regimen is a third-generation cephalosporin, such as cefotaxime or ceftazidime.^[11]^ These antibiotics have good activity against gram-negative bacteria and effectively treat SBP. Alternatively, a combination of a beta-lactam antibiotic with an agent that covers anaerobic bacteria, such as metronidazole, may be used.^[12]^ This combination is particularly useful in patients with risk factors for anaerobic infections, such as recent abdominal surgery or perforated viscus. For SBP, a course of antibiotics lasting 10 to 14 days is recommended.^[13]^ Our case antibiotics treatment followed the guidelines.

It is important to note that antibiotic selection should be tailored based on local antimicrobial resistance patterns and individual patient factors, such as allergy history and comorbidities.^[14]^ Additionally, once culture and sensitivity results are available, antibiotic therapy should be adjusted accordingly to optimize efficacy and minimize the risk of resistance development.^[15]^ Therefore, consultation with an infectious disease specialist may be beneficial in guiding antibiotic selection and management of bacterascites.^[16]^ Our case antibiotic choice was changed to meropenum after the culture result was determined.

SBP caused by Salmonella spp. is infrequent.^[17]^ Salmonella species in ascitic fluid following an emergency C/S can occur due to several potential factors, including preexisting colonization or infection, postoperative infection, hematogenous spread, postoperative complications, or immunocompromised state.^[17,18]^ Patients with conditions such as chronic hypertension or other comorbidities may have impaired immune function, making them more susceptible to infections, including those caused by Salmonella, like in our case.^[19]^

Table 1 reviews cases of SBP during pregnancy.^[20–24]^ It includes 6 cases with varying ages and delivery methods. Infections were caused by different bacteria: group A streptococci, staphylococcus aureus, *E coli*, Klebsiella, and Salmonella. Delivery methods included normal spontaneous delivery and C/S, with 1 case involving preterm labor and vaginal delivery after laparotomy. One case was dead within 12 hours of onset.^[20]^ The current case, involving a 41-year-old patient, developed SBP 3 days after a C/S due to a Salmonella infection.

**Table 1:** Literature review of spontaneous bacterial peritonitis occurring in pregnancy.

Author, yr	Age	Happen time and delivery method	Causing agents
Aziz Daghmouri et al 2020^[20]^	37	4 d after NSD, dead within 12 h	Group A streptococci
Dogra et al 2017^[21]^	21	Pregnancy 26 + 6 wk, received laparotomy, preterm labor and vaginal delivery at 30 wk	*Staphylococcus aureus*
Dhaliwal et al 2023^[22]^	20	9 d after NSD, laparotomy at admission d 8	Negative
Taniguchi et al 2011^[23]^	36	3 wk after embryo transfer, C/S at pregnancy 31 wk	*E coli*
Stauffer et al 1982^[24]^	22	Pregnancy 30 wk, emergent C/S at 32 wk	*Klebsiella and E coli*
Current case, 2024	41	3 d after C/S	*Salmonella*

C/S = cesarean section, NSD = normal spontaneous delivery.

In conclusion, Salmonella is rarely associated with bacterascites. Bacteroscites, in this case, may be associated with multiple causes, including hypoproteinemia from preeclampsia and allergic or inflammatory reactions of the peritoneum. Antibiotic therapy was the mainstay treatment. Paracentesis can ease the abdominal discomfort symptoms. Fluid supplements for hypoalbuminemia should be carefully monitored for pulmonary complications. Therefore, diuretics may be helpful in such situations.

## Author contributions

**Conceptualization:** Sheng-Po Kao, Dah-Ching Ding.

**Data curation:** Ya-Chu Wu, Wang-Chun Ho, Sheng-Po Kao, Dah-Ching Ding.

**Formal analysis:** Ya-Chu Wu, Wang-Chun Ho, Sheng-Po Kao, Dah-Ching Ding.

**Supervision:** Dah-Ching Ding.

**Writing – original draft:** Ya-Chu Wu, Wang-Chun Ho, Sheng-Po Kao, Dah-Ching Ding.

**Writing – review & editing:** Ya-Chu Wu, Dah-Ching Ding.
